# A160 A CASE OF BREAST CANCER METASTASIS MASQUERADING AS SIGNET RING CELL ADENOCARCINOMA OF THE STOMACH

**DOI:** 10.1093/jcag/gwad061.160

**Published:** 2024-02-14

**Authors:** E Stephenson, C Zeman-Pocrnich, M Sey

**Affiliations:** Department of Gastroenterology, London Health Sciences Centre, London, ON, Canada; Department of Pathology, London Health Sciences Centre, London, ON, Canada; Department of Gastroenterology, London Health Sciences Centre, London, ON, Canada

## Abstract

**Background:**

Gastric cancer has a poor prognosis, particularly at advanced stages. Endoscopic submucosal dissection (ESD) can be used to treat selected early-stage gastric cancers. This case study explores the challenge of diagnosing and managing patients when metastatic lesions mimic primary gastric cancer, as seen in this case of breast cancer metastasis resembling signet ring cell adenocarcinoma of the stomach.

**Aims:**

The aim of this case report is to describe an unusual case of breast cancer metastasis to the stomach identified unexpectedly on random gastric biopsies, initially diagnosed as a primary gastric signet ring cell adenocarcinoma.

**Methods:**

Clinical and pathological records of the described patient were reviewed retrospectively. Furthermore, a review of the relevant literature was also performed.

**Results:**

A 68 year-old-woman underwent gastroscopy for nausea and vomiting. Her past medical history was remarkable for bilateral mastectomies ten years ago for invasive lobular carcinoma, with metastatic bone disease diagnosed by imaging five years prior. She had been well controlled on letrozole taken intermittently due to side effects. At endoscopy, while the stomach appeared grossly normal, random biopsies done for Helicobacter pylori identified a single focus of signet ring cell adenocarcinoma. Subsequent chromoendoscopy identified an area of subtle mucosal abnormality for which the patient declined surgery and was instead managed by ESD which surprisingly yielded no dysplasia. A follow up gastroscopy with random biopsies also failed to identify any areas of dysplasia, but random biopsies on a subsequent gastroscopy again identified a minute focus of adenocarcinoma with signet ring cell morphology. Given the unusual presentation of apparent signet ring cell adenocarcinoma in two distinct areas in the stomach without any visible endoscopic abnormality an in-depth pathological analysis was conducted. By immunohistochemistry the tumor cells stained positive for Estrogen Receptor, GATA3, and CK7 and negative for antibodies targeting CK20 and CDX2, which supported an interpretation of minute multifocal breast cancer metastases to the stomach from hematogenous spread.

**Conclusions:**

In this case, repeated endoscopic investigations and immunohistochemical staining revealed minute multifocal breast cancer metastases to the stomach. Conventional tumor staging methods often cannot identify microscopic metastases, potentially impacting disease progression and patient care. A high level of suspicion for metastatic breast cancer and a detailed pathological analysis allowed for the correct diagnosis in this patient.

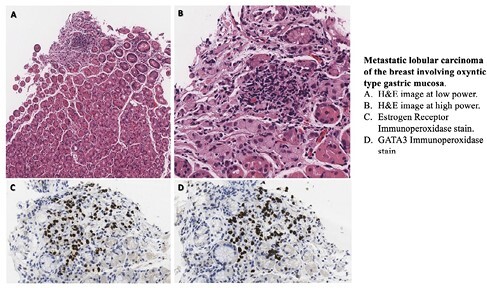

**Funding Agencies:**

None

